# Chronic Activation of Gp1 mGluRs Leads to Distinct Refinement of Neural Network Activity through Non-Canonical p53 and Akt Signaling

**DOI:** 10.1523/ENEURO.0438-19.2020

**Published:** 2020-03-27

**Authors:** Dai-Chi Liu, Stephanie Soriano, Yeeun Yook, Simon Lizarazo, Daphne E. Eagleman, Nien-Pei Tsai

**Affiliations:** 1 Neuroscience Program, University of Illinois at Urbana-Champaign, Urbana, IL 61801; 2 Department of Molecular and Integrative Physiology, School of Molecular and Cellular Biology, University of Illinois at Urbana-Champaign, Urbana, IL 61801

## Abstract

Group 1 metabotropic glutamate receptors (Gp1 mGluRs), including mGluR1 and mGluR5, are critical regulators for neuronal and synaptic plasticity. Dysregulated Gp1 mGluR signaling is observed with various neurologic disorders, including Alzheimer’s disease, Parkinson’s disease, epilepsy, and autism spectrum disorders (ASDs). It is well established that acute activation of Gp1 mGluRs leads to elevation of neuronal intrinsic excitability and long-term synaptic depression. However, it remains unknown how chronic activation of Gp1 mGluRs can affect neural activity and what molecular mechanisms might be involved. In the current study, we employed a multielectrode array (MEA) recording system to evaluate neural network activity of primary mouse cortical neuron cultures. We demonstrated that chronic activation of Gp1 mGluRs leads to elevation of spontaneous spike frequency while burst activity and cross-electrode synchronization are maintained at the baseline. We further showed that these neural network properties are achieved through proteasomal degradation of Akt that is dependent on the tumor suppressor p53. Genetically knocking down p53 disrupts the elevation of spontaneous spike frequency and alters the burst activity and cross-electrode synchronization following chronic activation of Gp1 mGluRs. Importantly, these deficits can be restored by pharmacologically inhibiting Akt to mimic inactivation of Akt mediated by p53. Together, our findings reveal the effects of chronic activation of Gp1 mGluRs on neural network activity and identify a unique signaling pathway involving p53 and Akt for these effects. Our data can provide insights into constitutively active Gp1 mGluR signaling observed in many neurologic and psychiatric disorders.

## Significance Statement

Group 1 metabotropic glutamate receptors (Gp1 mGluRs) are critical effectors of neuroplasticity and essential for neurodevelopment and cognition. Constitutively active Gp1 mGluR signaling has been observed in many pathologic conditions but the effects of chronic activation of Gp1 mGluRs on neuroplasticity are largely unknown. Our study provides evidence to demonstrate that chronic activation of Gp1 mGluRs refines the neural network properties through p53-dependent inactivation of Akt. These findings uncover an unconventional signaling pathway underlying Gp1 mGluR-dependent neuroplasticity and suggest distinct effects following versus chronic activation of Gp1 mGluRs.

## Introduction

Acute activation of metabotropic glutamate receptors (mGluRs) is known to trigger depression or elimination of the excitatory synapses (Nosyreva and Huber, 2006; Wilkerson et al., 2014). This is particularly pertinent for group 1 (Gp1) mGluRs because abnormal Gp1 mGluR signaling is frequently observed in neurodevelopmental and cognitive disorders such as fragile X syndrome (FXS), autism spectrum disorders (ASDs), and Alzheimer’s disease (Bear et al., 2004; Kleijer et al., 2014; Kumar et al., 2015), where synaptic strength or number is dysregulated. Gp1 mGluR-dependent synaptic depression and elimination are thought to be critical for circuit development (Takayasu et al., 2010), reward mechanism (Lüscher and Huber, 2010), and memory consolidation (Di Prisco et al., 2014). Despite these discoveries, our knowledge about the effects following chronic activation of Gp1 mGluRs is very limited. This is an important issue because constitutively active Gp1 mGluRs have been observed in FXS and several ASDs (Sharma et al., 2010; Ronesi et al., 2012; Lugo et al., 2014), and potentially contribute to hyperexcitability and elevated seizure susceptibility in those diseases (Levitt et al., 2004; Amaral et al., 2008; Fassio et al., 2011). Our current study is designed to address this issue.

To study the effects of chronic activation of Gp1 mGluR, we focus on neural network activity, a phenomenon that is less understood in regards to Gp1 mGluR signaling. Previous studies showed that acutely activating Gp1 mGluRs triggers oscillatory network activity in the mouse cortex and cortical neuron cultures (Hays et al., 2011; Liu et al., 2017). However, the effects following chronic activation of Gp1 mGluRs are unknown. To this end, we employed a multielectrode array (MEA) recording system and characterized that chronic activation of Gp1 mGluRs leads to elevation of spontaneous spike frequency while spike amplitude, cross-electrode synchronization and burst activity are maintained at the baseline. These findings revealed the role of chronic Gp1 mGluR activation on neural network activity.

Evidence suggests that Gp1 mGluR-mediated neuroplasticity mechanisms often require phosphatidylinositol-4,5-bisphosphate 3-kinase (PI3K) or extracellular signal-regulated kinase-1/2 (ERK1/2) signaling (Kinkl et al., 2001; Liu et al., 2013; Wang et al., 2014), which leads to S6K-dependent initiation of protein translation (Banko et al., 2006; Aguilar-Valles et al., 2015). In addition to protein translation, a study has also shown potential interplay between transcription and translation on Gp1 mGluR activation (Korb and Finkbeiner, 2011). However, it is unknown what transcription factors may be involved. To begin to search for the signaling pathways downstream of chronic activation of Gp1 mGluRs, we are studying murine double minute-2 (Mdm2), a ubiquitin EIII ligase which was recently shown to participate in Gp1 mGluR-induced protein translation (Liu et al., 2017). Our current study showed that Mdm2 is downregulated on chronic activation of Gp1 mGluRs. Mdm2 is a well-characterized ubiquitin EIII ligase in the field of cancer biology, and the tumor suppressor p53 is one of its major substrates (Devine and Dai, 2013). Indeed, we observed elevated p53 following chronic activation of Gp1 mGluRs in cortical neuron cultures. Furthermore, we found that elevation of p53 leads to proteasomal degradation of Akt. This Mdm2-p53-Akt signaling represents a unique pathway for Gp1 mGluR-dependent plasticity mechanisms. Mdm2-p53 signaling had not been shown to regulate neuronal plasticity until recently (Jewett et al., 2015, 2016; Lisachev et al., 2015; Pustylnyak et al., 2015), and still little is known about p53 signaling in the central nervous system. Our current study introduces a novel function of p53 in the regulation of activity-dependent neural network plasticity. With the deep knowledge of Mdm2-p53 in cancer biology, our research could rapidly facilitate the study of, or even future treatment for, neurologic diseases associated with constitutively active Gp1 mGluR signaling.

## Materials and Methods

### Mice

The *p53^flox^* and *Emx1*-Cre mice in C57BL/6J background were obtained from The Jackson Laboratory. Trio breeding was conducted throughout the study. Both male and female mice were used to prepare mixed sex cultures. All animal procedures were performed in accordance with our institutional animal care committee’s regulations. For genotyping, the primers used to detect *p53 loxP* allele are: 5’-GGTTAAACCCAGCTTGACCA-3′, and 5’-GGAGGCAGAGACAGTTGGAG-3′. The primers used to differentiate *wild-type* and *Emx1* alleles are 5’-CGG TCT GGC AGT AAA AAC TAT C-3’ (*Emx1-Cre*), 5’-GTG AAA CAG CAT TGC TGT CAC TT-3’ (*Emx1-Cre*); 5’-AAG GTG TGG TTC CAG AAT CG-3’ (*wild-type*), and 5’-CTC TCC ACC AGA AGG CTG AG-3’ (*wild-type*).

### Reagents

DHPG was from Abcam. Dimethyl sulfoxide (DMSO) and MG132 were from Fisher Scientific. MK-2206 was from AdooQ Bioscience. DMSO was used as the vehicle throughout this study. The antibodies used in this study were purchased from Santa Cruz Biotechnology (anti-Mdm2), GenScript Corporation (anti-Gapdh), and Cell Signaling (anti-Akt, anti-p-Akt, anti-Erk1/2, anti-p-Erk1/2, anti-p53, and anti-ubiquitin). Horseradish peroxidase (HRP)-conjugated secondary antibodies were from Santa Cruz Biotechnology, Cell Signaling, and Jackson ImmunoResearch.

### Primary neuron cultures

Primary neuron cultures were made from mice aged at p0–p1 and maintained in Neural Basal A medium supplemented with B27 supplement (Invitrogen), GlutaMax (final concentration at 2 mM; Invitrogen), and cytosine β-D-arabinofuranoside (AraC, final concentration at 2 μM; Sigma). The culture medium was changed 50% on DIV2 and every 3–4 d thereafter until the experiments on DIV14.

### MEA recording

All the MEA recordings were performed using an Axion Muse 64-channel system in single-well MEAs (M64-GL1-30Pt200, Axion Biosystems) inside a 5% CO_2_, 37°C, and humidified incubator. Field potentials (voltage) at each electrode relative to the ground electrode were recorded with a sampling rate of 25 kHz. After 30 min of recording the baseline (before treatment), drug(s) or vehicles indicated in each experiment were added, and the MEA dish was immediately put back into the incubator for 24 h before another 30 min of recording (after treatment). Because of changes in network activity caused by physical movement of the MEA, only the last 15 min of each recording were used in data analyses (Zhu et al., 2017). AxIS software (Axion Biosystems) was used for the extraction of spikes (i.e., action potentials) from the raw electrical signal obtained from the Axion Muse system. After filtering, a threshold of ±6 SD, as also used by others in the past (Speer et al., 2014; Newberry et al., 2016; Nik et al., 2017; Lewandowska et al., 2018; Sarkar et al., 2018; Alarautalahti et al., 2019; Quraishi et al., 2019; Sun et al., 2019; Negri et al., 2020), was independently set for each channel; activity exceeding this threshold was counted as a spike. The settings for burst detection in each electrode were a minimum of five spikes with a maximum interspike interval of 0.1 s (Zhu et al., 2017). The burst duration, number of spikes per burst, and interburst interval were analyzed by AxIS software. Synchrony index was also computed through AxIS software, based on a published algorithm (Eggermont, 2006), by taking the cross-correlation between any two spike trains, removing the portions of the cross-correlogram that are contributed by the auto-correlations of each spike train, and reducing the distribution to a single metric. For all before and after drug treatment comparisons, to minimize the variability between cultures, the recording from each MEA culture after treatment was compared with the baseline recording from that same culture.

### Immunoprecipitation (IP) and Western blotting

For IP, cell lysates were obtained by sonicating pelleted cells in IP buffer (50 mM Tris, pH 7.4, 120 mM NaCl, and 0.5% Nonidet P-40); 80 μg of total protein mixtures was incubated for 1 h at 4°C with 0.5-μg primary antibodies. Protein A/G agarose beads were added for another hour followed by washing with IP buffer three times. For Western blotting, after SDS-PAGE, the gel was transferred onto a polyvinylidene fluoride membrane (Santa Cruz Biotechnology). After blocking with 1% bovine serum albumin in TBST buffer (20 mM Tris, pH 7.5, 150 mM NaCl, and 0.1% Tween 20), the membrane was incubated with primary antibody overnight at 4°C, followed by three 10-min washings with TBST buffer. The membrane was then incubated with an HRP-conjugated secondary antibody for 1 h at room temperature, followed by another three 10-min washings. Finally, the membrane was developed with an ECL Chemiluminescent Reagent. All the Western blotting results were semi-quantitatively normalized to the control groups before statistical analysis.

### Real-time quantitative reverse transcription PCR (RT-qPCR)

After drug treatment, the total RNA from cortical neurons in cultures was obtained with TRIzol reagent (Life Technologies). Reverse transcription was performed with Photoscript reverse transcriptase (New England Biolab) and the real-time PCR was performed with Thermo Scientific Maxima SYBR Green reagent. The primers used in this study were: Akt (pair 1), 5’-AAC GGA CTT CGG GCT GTG-3’ and 5’-TTG TCC TCC AGC ACC TCA GG-3’; Akt (pair 2), 5’-AGA AGA GAC GAT GGA CTT CCG-3’ and 5’-TCA AAC TCG TTC ATG GTC ACA C-3’; Actin, 5’-CCT GTG CTG CTC ACC GAG GC-3’ and 5’-GAC CCC GTC TCT CCG GAG TCC ATC-3’.

### Quantitative Akt activity assay

The assay was conducted using Akt kinase assay kit from Enzo Life Sciences. In brief, after drug treatment, the cell lysates were obtained from cultures by light sonication followed by an ELISA analysis using a 96-well plate coated with a specific synthetic peptide as a substrate for Akt. The activity of Akt was determined by the level of phosphorylation on the peptide, detected by a phospho-specific antibody. With the use of a HRP-conjugated secondary antibody, the signaling was developed using 3,3′,5,5′-tetramethylbenzidine (TMB) and measured in a microplate reader at 450 nm.

### Cell viability assay

The assay was conducted using Cell Counting kit–8 from Sigma. In brief, primary cortical neuron cultures on a 96-well plate were added with (2-(2-methoxy-4-nitrophenyl)−3-(4-nitrophenyl)−5-(2,4-disulfophenyl)-2H-tetrazolium, monosodium salt), or called WST-8. WST-8 can be reduced by dehydrogenases in viable cells and transformed to formazan, an orange colored dye soluble in the culture medium. The number of viable cells can therefore be determined by the relative intensity of the orange color measured in a microplate reader at 450 nm.

### Statistical analysis

The data presented in this study have been tested for normality using Kolmogorov–Smirnov test. Statistical methods to determine significance along with sample numbers were indicated in each figure legend. In brief, ANOVA with *post hoc* Tukey HSD test was used for multiple comparisons between treatments or genotypes. Student’s *t* test was used for paired samples and one sample *t* test was used for conditions where experimental groups are normalized to control groups. Each *n* indicates an independent culture. Differences are considered significant at the level of *p *<* *0.05.

## Results

### Chronic activation of Gp1 mGluRs elevates spontaneous network activity

Acute activation of Gp1 mGluR leads to elevation of neural network activity (Liu et al., 2017). Despite the fact that Gp1 mGluRs are involved in the onset of long-term epilepsy, how chronic activation of Gp1 mGluRs affects neural network activity remains unclear. To study and activate Gp1 mGluRs, we treated wild-type (WT) primary cortical neuron cultures with the (RS)−3,5-dihydroxyphenylglycine (DHPG; 100 μM) or vehicle (DMSO) starting on day *in vitro* (DIV)13–DIV14, as previously described (Zhu et al., 2017; Liu et al., 2019). To first determine the effectiveness of DHPG during chronic administration, we collect culture medium from cultures continuously treated with DHPG for 0, 6, 12, and 24 h without washout. We subsequently applied the medium onto fresh cultures for 30 min, followed by Western blotting to detect phosphorylation status of ERK, an indication of acute Gp1 mGluR activation (Ronesi and Huber, 2008). As shown (Extended Data Fig. 1-1), the activity of DHPG remained strong after 6 h but was back to the baseline after 12–24 h. These data suggest DHPG remains active in cultures for at least 6 h. Based on these results, we chose 24 h as our time point to study potential plasticity following chronic activation of Gp1 mGluR activation and at the same time to mimic the washout when DHPG is no longer active, as done previously (Bianchi et al., 2009).

**Figure 1.**
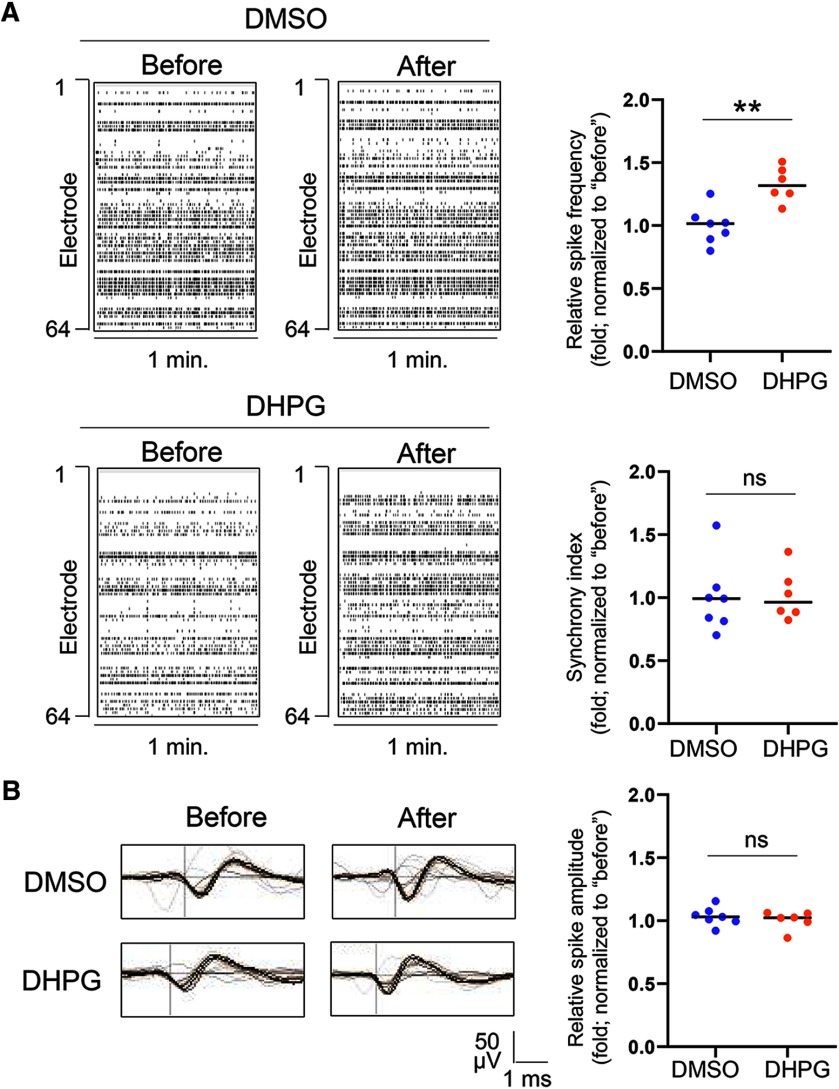
Spontaneous spike frequency is elevated after chronic activation of Gp1 mGluRs. ***A***, Raster plots of spontaneous spikes from representative 1-min recordings of WT cortical neuron cultures treated with vehicle (DMSO) or DHPG (100 μM) for 24 h at DIV14. Quantification of relative spontaneous spike frequency and synchrony index by comparing “after treatment” to “before treatment” of the same cultures during the 15-min recordings is on the right. ***B***, Representative average traces of spike amplitude from 1-min recordings of WT cortical neuron cultures treated with DMSO or DHPG (100 μM) for 24 h at DIV14. In the traces, the black lines represent the average of all the spikes within representative 1-min recordings. Traces are from the same designated electrodes before and after treatments. Quantification of spike amplitude is done by comparing “after treatment” to “before treatment” during the 15-min recordings from the same cultures; *n* = 7 and *n* = 6 independent cultures for DMSO and DHPG treatment groups, respectively. Student’s *t* test was used. Data are represented as mean ± SEM with **p < 0.01, ns: non-significant.

10.1523/ENEURO.0438-19.2020.f1-1 Extended Data Figure 1-1 Time course of DHPG activity in cultures. Representative Western blottings of ERK, p-ERK, and Gapdh, and their quantification from WT cortical neuron cultures treated with conditional medium collected from cultures that were administered with DHPG for 0, 6, 12, or 24 h (*n* = 4). Download Figure 1-1, TIF file

To study neural network activity, we employed an MEA system to record extracellular spontaneous spikes (action potentials). The recordings were performed immediately before and 24 h after treatment. When comparing the recordings from the same cultures before and after treatment, we found that the frequency of spontaneous spikes is significantly elevated after continuous 24 h of DHPG administration (Fig. 1*A*). We also compared the neural network synchrony after the treatment of DHPG and DMSO for 24 h by quantification of the synchrony index, which is based on the synchrony of spike firing between electrode pairs throughout the entire MEA. As shown (Fig. 1*A*), the neural network synchrony does not differ between DHPG and DMSO treated groups. In addition, the average spontaneous spike amplitude did not differ between two treatment groups either (Fig. 1*B*). These data conclude that chronic activation of Gp1 mGluRs leads to an elevation of spontaneous spike frequency.

**Figure 2.**
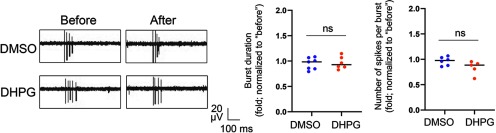
Burst activity is at the baseline after chronic activation of Gp1 mGluRs. Representative traces of burst activity from WT cortical neuron cultures treated with DMSO or DHPG (100 μM) for 24 h at DIV14. Traces are from the same designated electrode “before” and “after” drug treatments. Quantification of burst duration and relative number of spikes per burst by comparing “after treatment” to “before treatment” during the 15-min recordings from the same cultures is on the right; *n* = 7 and *n* = 6 independent cultures for DMSO and DHPG treatment groups, respectively. Student’s *t* test was used. Data are represented as mean ± SEM with ns: non-significant.

In addition to elevated spontaneous spike frequency, we also asked whether the pattern of spontaneous spikes is affected by chronic activation of Gp1 mGluRs. This is based on a previous study showing an elevation of burst activity, which is a series of spontaneous spikes occurred within a short period of time, following acute activation of Gp1 mGluRs (Liu et al., 2017). We defined a minimum of five spontaneous spikes with a maximum interspike interval of 0.1 s as a burst, based on a previous study (Zhu et al., 2017), and found that the burst activity, demonstrated by burst duration and average number of spikes per burst, does not differ between DHPG and DMSO treated groups (Fig. 2). Because acute activation of Gp1 mGluRs is previously known to robustly elevate burst activity (Liu et al., 2017), our results suggest that either a desensitization or a homeostatic reduction of burst firing after chronic activation of Gp1 mGluRs is likely occurring.

### Chronic activation of Gp1 mGluRs leads to proteasome-mediated downregulation of Akt

**Figure 3.**
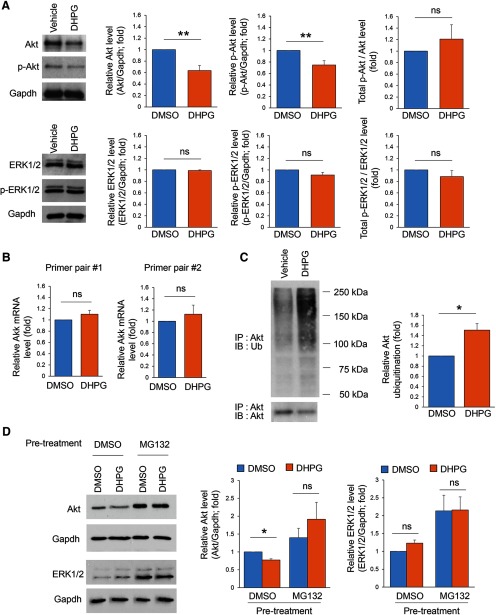
Chronic activation of Gp1 mGluRs leads to proteasomal degradation of Akt. ***A***, Representative Western blottings of Akt, p-Akt, ERK, p-ERK, and Gapdh, and their quantification from WT cortical neuron cultures treated with DMSO or DHPG (100 μM) for 24 h at DIV14 (*n* = 8 and *n* = 9 sets of cultures for detecting Akt and ERK, respectively). ***B***, RT-qPCR of Akt mRNA, using two different pairs of primers, normalized to Actin mRNA from WT cortical neuron cultures treated with DMSO or DHPG (100 μM) for 24 h at DIV14 (*n* = 4). ***C***, Representative Western blottings of Ubiquitin and Akt after IP with anti-Akt antibody using lysates from WT cortical neuron cultures treated with DMSO or DHPG (100 μM) for 24 h at DIV14 (*n* = 4). ***D***, Representative Western blottings of Akt, ERK, and Gapdh and the quantification from WT cortical neuron cultures treated with DMSO, DHPG (100 μM), MG132 (10 μM), and DHPG+MG132 at DIV14 (*n* = 4). MG132 was applied during the second 12 h of DMSO or DHPG treatments. For the quantification above, Student’s *t* test (***A–C***) or a two-way ANOVA with Tukey’s test (***D***) were used. Data are represented as mean ± SEM with *p < 0.05, **p < 0.01, ns: non-significant.

We next aim to determine whether a particular signaling pathway contributes to the neural network properties that we observed following chronic activation of Gp1 mGluRs in Figures 1, 2. It is widely accepted that acute activation of Gp1 mGluRs activates two downstream signaling pathways: MEK-ERK and PI3K-Akt pathways (Ronesi and Huber, 2008). To this end, we assessed the activity of ERK and Akt by quantifying the total protein levels and phosphorylation status of ERK and Akt after treatments of DMSO or DHPG for 24 h. As shown (Fig. 3*A*), we observed a significant downregulation of total protein levels and phosphorylation of Akt, but not ERK, after DHPG treatments. This result was also supported by a quantitative Akt activity assay showing reduced total Akt enzymatic activity following DHPG treatment (Extended Data Fig. 3-1). Because the specific activity of Akt, demonstrated by the ratio of p-Akt to total Akt, does not differ between DHPG and DMSO treated groups (Fig. 3*A*, right), it suggests that chronic activation of Gp1 mGluRs leads to inactivation of Akt signaling through downregulation of Akt proteins.

10.1523/ENEURO.0438-19.2020.f3-1 Extended Data Figure 3-1 DHPG treatment for 24 h triggers p53-dependent reduction of Akt kinase activity. Quantification of Akt kinase activity from DMSO- or DHPG-treated *p53^f/+^*^−^*^Emx1^*^-Cre−^ or *p53^f/+^*^−^*^Emx1^*^-Cre+^ cortical neuron cultures (*n* = 6). A two-way ANOVA with Tukey’s test was used. Data are represented as mean ± SEM with *p < 0.05, ns: non-significant. Download Figure 3-1, TIF file.

To determine whether and how chronic activation of Gp1 mGluRs modulates Akt protein levels, we first measure the levels of Akt mRNA in WT cortical neuron cultures following the treatment of DHPG or DMSO for 24 h. We performed real-time reverse transcription and quantitative PCR (real-time RT qPCR) to measure the relative levels of Akt mRNA with two independent pairs of primers. As shown (Fig. 3*B*), cultures treated with DHPG or DMSO exhibit similar levels of Akt mRNA, suggesting the effect of chronic activation of Gp1 mGluRs on downregulation of Akt is unlikely to occur at the stage of transcription. Akt is known to be regulated by ubiquitination and proteasome-mediated degradation (Chan et al., 2012). To determine whether the downregulation of Akt occurs through protein ubiquitination and subsequent degradation, we performed IP with anti-Akt antibody followed by Western blotting with anti-Ubiquitin antibody to assess Akt ubiquitination. As shown (Fig. 3*C*), ubiquitination of Akt is elevated in cultures treated with DHPG. This result indicated a possibility that Akt is degraded in proteasome following treatment of DHPG. To confirm this possibility, as shown (Fig. 3*D*), we found that treatment of a proteasome inhibitor MG132 (10 μM) during the last 12 h of DHPG treatment efficiently inhibits the downregulation of Akt. Although ERK was not altered after treatment of DHPG (Fig. 3*A*), its level was elevated following treatment of MG132 (Fig. 3*D*), indicating ERK can also be regulated by proteasome. Altogether, our results show that chronic activation of Gp1 mGluRs leads to downregulation and inactivation of Akt through proteasome-dependent downregulation of Akt.

### Chronic activation of Gp1 mGluRs inhibits Mdm2-p53 signaling pathway

**Figure 4.**
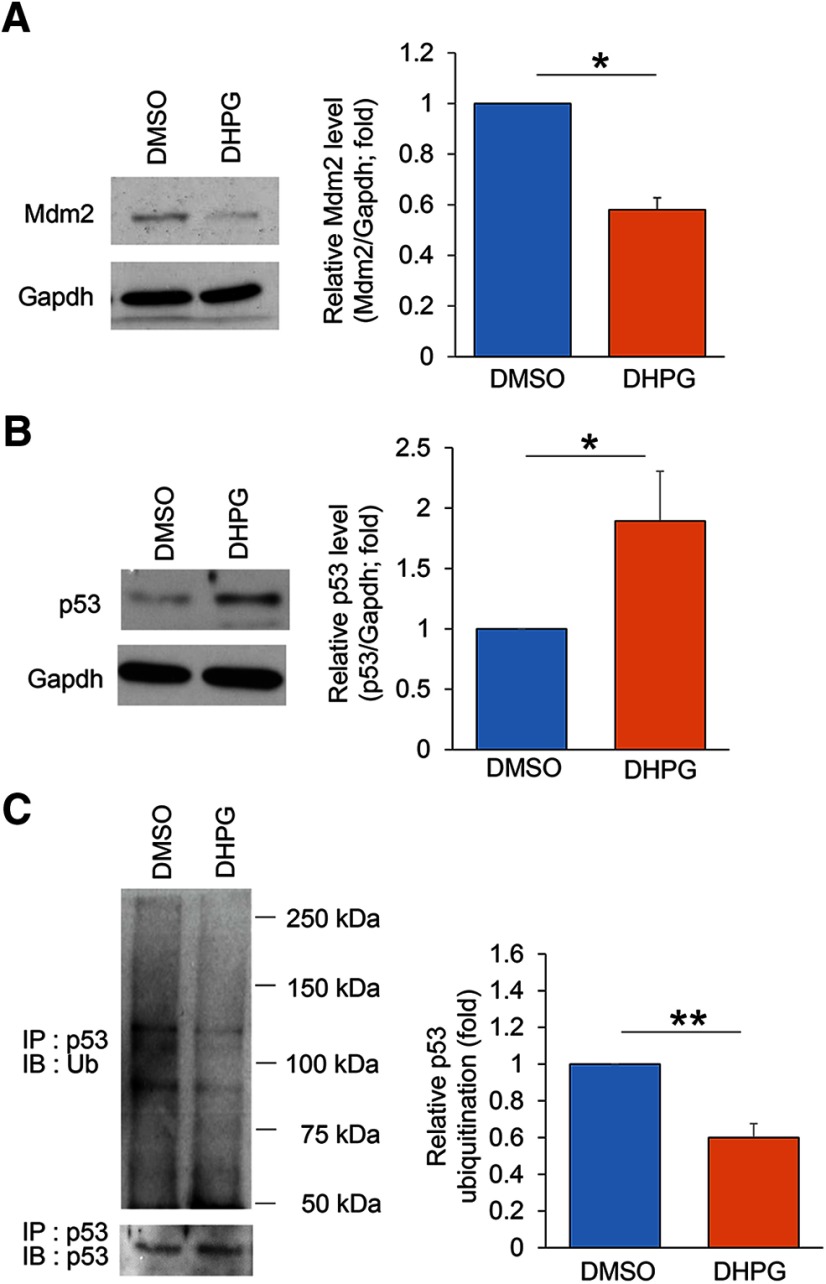
Chronic activation of Gp1 mGluRs reduces Mdm2-p53 signaling. ***A***, Representative Western blottings of Mdm2, and Gapdh from WT cortical neuron cultures treated with DMSO or DHPG (100 μM) for 24 h at DIV14 (*n* = 4). ***B***, Representative Western blottins of p53, and Gapdh from WT cortical neuron cultures treated with DMSO or DHPG (100 μM) for 24 h at DIV14 (*n* = 8). ***C***, Representative Western blottings of Ubiquitin and p53 after IP with anti-p53 antibody using lysates from WT cortical neuron cultures treated with DMSO or DHPG (100 μM) for 24 h at DIV14 (*n* = 3). Student’s *t* test was used. Data are represented as mean ± SEM with *p < 0.05, **p < 0.01.

We next search for the upstream signaling pathway that mediates downregulation of Akt following chronic activation of Gp1 mGluRs. It has been shown previously that acute activation of Gp1 mGluRs downregulates the ubiquitin EIII ligase, Mdm2 (Liu et al., 2017). To determine whether Mdm2 is regulated similarly on chronic activation of Gp1 mGluRs, we measured the total protein levels of Mdm2 and observed significant downregulation of Mdm2 following DHPG treatment for 24 h (Fig. 4*A*). Mdm2 is known to ubiquitinate its substrate tumor suppressor p53 (Jewett et al., 2018; Lee et al., 2018). Because Mdm2 is downregulated, we asked whether p53 is elevated following chronic activation of Gp1 mGluRs. As shown (Fig. 4*B*), the total protein levels of p53 are indeed significantly elevated following DHPG treatment for 24 h. Consistently, a reduction of p53 ubiquitination was also observed following DHPG treatment for 24 h (Fig. 4*C*). These results confirmed a reduction of Mdm2 and an elevation of p53 on chronic activation of Gp1 mGluRs.

### p53 mediates chronic activation of Gp1 mGluR-induced downregulation of Akt

**Figure 5.**
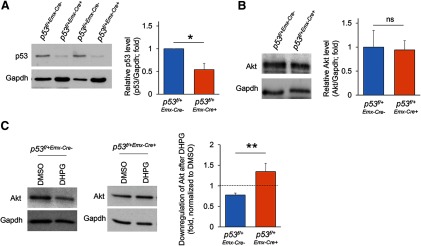
p53 is required for chronic activation of Gp1 mGluRs-induced downregulation of Akt. ***A***, Quantification of p53 expression and representative Western blottings of p53 and Gapdh from *p53^f/+^*^-^*^Emx1^*^-Cre+^ or *p53^f/+^*^-^*^Emx1^*^-Cre+;^ cortical neuron cultures at DIV14 (*n* = 3). ***B***, Quantification of Akt expression and representative Western blottings of p53 and Gapdh from *p53^f/+^*^-^*^Emx1^*^-Cre+^ or *p53^f/+^*^-^*^Emx1^*^-Cre+^ cortical neuron cultures at DIV14 (*n* = 3). ***C***, Quantification of Akt expression and representative Western blottings of Akt and Gapdh from *p53^f/+^*^-^*^Emx1^*^-Cre+^ or *p53^f/+^*^-^*^Emx1^*^-Cre+^ cortical neuron cultures treated with DMSO or DHPG (100 μM) for 24 h at DIV14 (*n* = 7 and *n* = 9 for p53 *p53^f/+^*^-^*^Emx1^*^-Cre+^ or *p53^f/+^*^-^*^Emx1^*^-Cre+^ cultures, respectively). Student’s t test was used. Data are represented as mean ± SEM with *p < 0.05, **p < 0.01, ns: non-significant.

In order to determine whether p53 is responsible for the downregulation of Akt following chronic activation of Gp1 mGluRs, we generate a conditional p53 knock-down mouse model by crossing p53 floxed mice (*p53*^f/f^) with *Emx1*-Cre mice to obtain *p53^f/+-Emx-Cre+^* and *p53^f/+-Emx-Cre−^*mice. *Emx1*-Cre can confer p53 reduction in the cortex and hippocampus, primarily in excitatory neurons, beginning as early as embryonic day (E)10.5 (Gorski et al., 2002; Young et al., 2007). We used heterozygous mice (*p53^f/+^*) to minimize potential effects on apoptosis mediated by complete p53 knock-out (KO; Fortin et al., 2001). The knock-down efficiency of p53 in *p53^f/+-Emx-Cre+^* cortical neuron cultures at DIV14 is ∼46% in comparison to the *p53^f/+^*^−^*^Emx1^*^-Cre−^ cultures, when cultures are prepared on postnatal day 0 (Fig. 5*A*). We first determine whether total Akt levels are different between two genotypes and found no difference (Fig. 5*B*). Further, despite much lower p53 at the basal levels, an elevation of p53 following DHPG treatment for 24 h is still apparent in *p53^f/+-Emx-Cre+^* cultures (Extended Data Fig. 5-1), suggesting that floxed-p53 allele does not alter the response to chronic activation of Gp1 mGluRs. We then determined whether chronic activation of Gp1 mGluRs-induced downregulation of Akt is impaired when p53 is knocked down in *p53^f/+-Emx-Cre+^*cortical neuron cultures. As shown (Fig. 5*C*), the total protein levels of Akt are downregulated in *p53^f/+-Emx-Cre−^*cultures after DHPG treatment for 24 h, as we observed in WT (C57BL/6J) cultures (Fig. 3*A*), but not in *p53^f/+-Emx-Cre+^* cultures. These data suggest that p53 is required for the reduction of Akt on chronic activation of Gp1 mGluRs.

10.1523/ENEURO.0438-19.2020.f5-1 Extended Data Figure 5-1 DHPG treatment for 24 h elevates p53 in *p53^f/+^*^−^*^Emx1^*^-Cre+^ cultures. Representative Western blottings of p53 and Gapdh from *p53^f/+^*^−^*^Emx1^*^-Cre+^ cortical neuron cultures treated with DMSO or DHPG (100 μM) for 24 h at DIV14 (*n* = 4). Student’s *t* test was used. Data are represented as mean ± SEM with *p < 0.05. Download Figure 5-1, TIF file.

### Pharmacological inhibition of Akt normalizes aberrant neural network activity following chronic activation of Gp1 mGluRs in p53 conditional knock-down cultures

**Figure 6.**
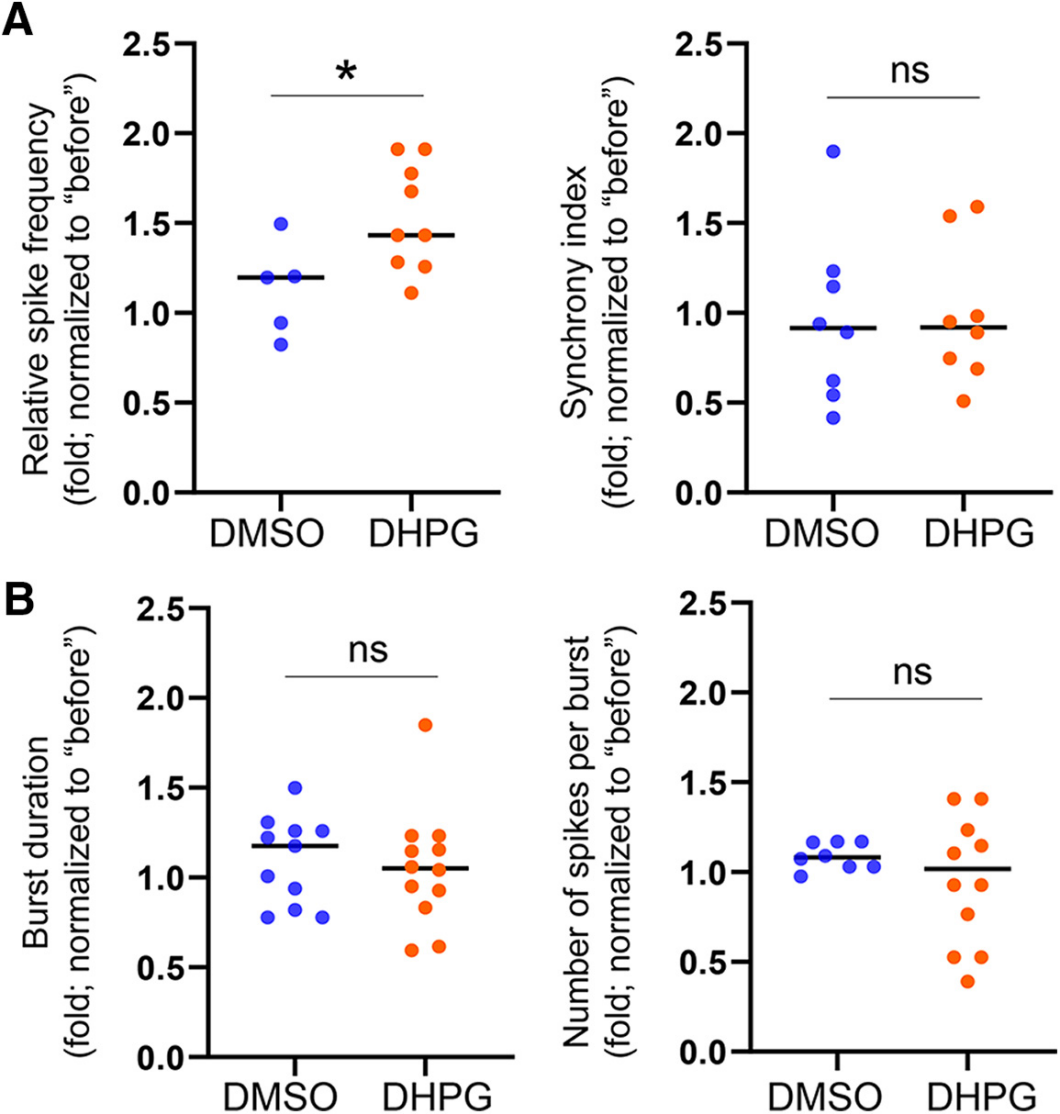
Chronic activation of Gp1 mGluRs elicits effects on neural network activity in *p53^f/+^*^-^*^Emx1^*^-Cre-^ cortical neuron cultures similar to those in WT (C57BL/6J) cortical neuron cultures. ***A***, ***B***, Quantification of relative spontaneous spike frequency (***A***, left), synchrony (***A***, right), burst duration (***B***, left) and relative number of spikes per burst (***B***, right) from *p53^f/+^*^-^*^Emx1^*^-Cre-^ cortical neuron cultures treated with vehicle (DMSO) or DHPG (100 μM) for 24 h at DIV14. The analysis was done by comparing “after treatment” to “before treatment” of the same cultures during the 15-min recordings. Student’s *t* test was used. Data are represented as mean ± SEM with *p < 0.05, ns: non-significant.

**Figure 7.**
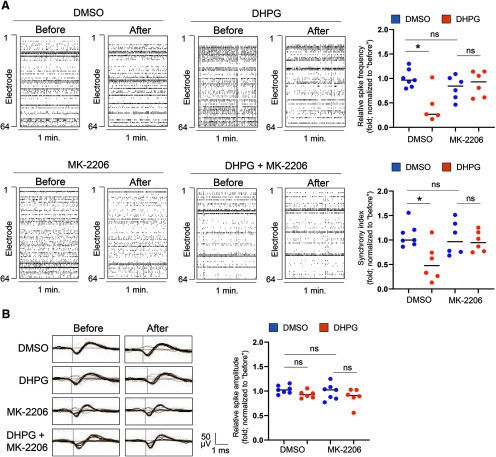
Pharmacological inhibition of Akt partially restores spontaneous network activity in *p53^f/+^*^-^*^Emx1^*^-Cre+^ cortical neuron cultures. ***A***, Raster plots of spontaneous spikes from representative 1-min recordings of *p53^f/+^*^-^*^Emx1^*^-Cre+^ cortical neuron cultures treated with DMSO, DHPG (100 μM), DMSO+MK-2206 (1 μM), or DHPG+MK-2206 at DIV14. Quantification of relative spontaneous spike frequency and synchrony index by comparing “after treatment” to “before treatment” of the same cultures during the 15-min recordings (*n* = 5–7 independent cultures). ***B***, Representative average traces of spike amplitude from 1-min recordings of WT cortical neuron cultures treated with DMSO, DHPG (100 μM), DMSO+MK-2206 (1 μM), or DHPG+MK-2206 at DIV14. In the traces, the black lines represent the average of all the spikes within representative 1-min recordings. Traces are from the same designated electrodes before and after treatments (*n* = 5–7 independent cultures). Quantification of spike amplitude is done by comparing “after treatment” to “before treatment” during the 15-min recordings from the same cultures. For both ***A***, ***B***, MK-2206 was applied during the last 1 h of 24-h DHPG or DMSO treatments. A two-way ANOVA with Tukey’s test was used. Data are represented as mean ± SEM with *p < 0.05, ns: non-significant.

We have now confirmed a reduction of Akt activity following chronic activation of Gp1 mGluRs through Mdm2-associated elevation of p53. To determine whether p53 is required for the neural network properties that we observed following chronic activation of Gp1 mGluRs (Figs. 1, 2), we aim to perform the same MEA recording in *p53^f/+-Emx-Cre+^* cultures, in which *p53* is knocked down. As a control, we first confirmed that the network activity following chronic activation of Gp1 mGluRs through the treatment of DHPG for 24 h in *p53^f/+-Emx-Cre−^* cultures is similar to what we observed in WT (C57BL/6J) cultures, in which spontaneous spike frequency is elevated while synchrony and burst activity are at the baseline (Fig. 6). These data support our previous data (Extended Data Fig. 5-1) that the floxed-p53 allele does not alter the response to chronic activation of Gp1 mGluRs. We then performed the same recording in *p53^f/+-Emx-Cre+^* cultures and found that chronic activation of Gp1 mGluRs surprisingly leads to a downregulation of spontaneous spike frequency (Fig. 7*A*). When compared the neural network synchrony, we also observed a downregulation of network synchrony after the treatment of DHPG for 24 h in *p53^f/+-Emx-Cre+^* cultures. To address the concern of whether such an effect is due to reduced viability of *p53^f/+-Emx-Cre+^* neurons, we performed viability assay and found no significant effects on the number of living cells following chronic treatments of DHPG (Extended Data Fig. 7-1). These data, together with our results in Figure 1, suggest that p53 is required for maintaining the spontaneous spike frequency and neural network synchrony following chronic activation of Gp1 mGluRs.

10.1523/ENEURO.0438-19.2020.f7-1 Extended Data Figure 7-1 DHPG treatment for 24 h does not affect cell viability. Quantification of cell viability from DMSO- or DHPG-treated *p53^f/+^*^−^*^Emx1^*^-Cre−^ or *p53^f/+^*^−^*^Emx1^*^-Cre+^ cortical neuron cultures (*n* = 5). A two-way ANOVA with Tukey’s test was used. Data are represented as mean ± SEM with ns: non-significant. Download Figure 7-1, TIF file.

Because chronic activation of Gp1 mGluRs-induced downregulation of Akt is absent in *p53^f/+-Emx-Cre+^* cultures (Fig. 5*B*), we asked whether excessive Akt activity is leading to this abnormal downregulation of spontaneous spike frequency and synchronization. To answer this question, we treated *p53^f/+-Emx-Cre−^* or *p53^f/+-Emx-Cre+^* cultures with a specific Akt inhibitor, MK-2206 (1 μM in DMSO), during the last 1 h of 24-h DHPG or DMSO treatments. As shown (Fig. 7*A*; Extended Data Fig. 7-2), although MK-2206 treatment did not elicit any significant effects toward basal or DHPG-induced changes in neural network activity in *p53^f/+-Emx-Cre−^* cultures (Extended Data Fig. 7-2*A*), MK-2206 treatment was able to inhibit the downregulation of spontaneous spike frequency as well as neural network synchrony following chronic activation of Gp1 mGluRs in *p53^f/+-Emx-Cre+^* cultures (Fig. 7*A*). The average spontaneous spike amplitude was not affected by any of the treatments (Fig. 7*B*). Together, these data confirmed that p53-mediated inactivation of Akt is required, at least partially, to maintain spontaneous spike frequency and neural network synchrony following chronic activation of Gp1 mGluRs.

10.1523/ENEURO.0438-19.2020.f7-2 Extended Data Figure 7-2 Inhibition of Akt does not affect basal neural network activity in control, *p53^f/+^*^−^*^Emx1^*^-Cre−^, cultures. ***A***, ***B***, Quantification of relative spontaneous spike frequency (***A***, left), synchrony (***A***, right), burst duration (***B***, left) and relative number of spikes per burst (***B***, right) from *p53^f/+^*^−^*^Emx1^*^-Cre−^ cortical neuron cultures treated with DMSO, DHPG (100 μM), DMSO+MK-2206 (1 μM), or DHPG+MK-2206 at DIV14. The analysis was done by comparing “after treatment” to “before treatment” of the same cultures during the 15-min recordings (*n* = 5–7 independent cultures). A two-way ANOVA with Tukey’s test was used. Data are represented as mean ± SEM with *p < 0.05 and ns: non-significant. Download Figure 7-2, TIF file.

**Figure 8.**
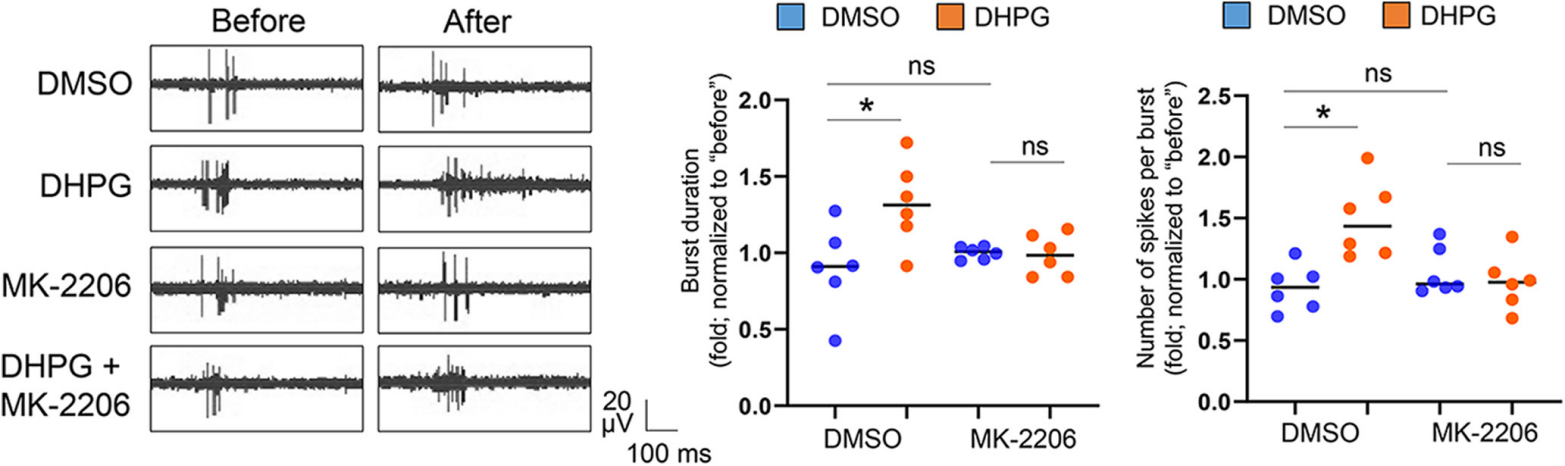
Aberrant burst activity in *p53^f/+^*^-^*^Emx1^*^-Cre+^ cortical neuron cultures is normalized by pharmacological inhibition of Akt. Representative traces of burst activity from *p53^f/+^*^-^*^Emx1^*^-Cre+^ cortical neuron cultures treated with DMSO, DHPG (100 μM), DMSO+MK-2206 (1 μM), or DHPG+MK-2206 at DIV14. MK-2206 was applied during the last 1 h of 24-h DHPG or DMSO treatments. Traces are from the same designated electrode “before” and “after” drug treatments. Quantification of burst duration and relative number of spikes per burst by comparing “after treatment” to “before treatment” during the 15-min recordings from the same cultures was on the right (*n* = 6 independent cultures). A two-way ANOVA with Tukey’s test was used. Data are represented as mean ± SEM with *p < 0.05, ns: non-significant.

Next, we asked how burst activity is affected when p53 is knocked down in *p53^f/+-Emx-Cre+^* cultures. We measured the burst duration and average number of spikes per burst in *p53^f/+-Emx-Cre−^* or *p53^f/+-Emx-Cre+^* cultures following the treatment of DHPG or DMSO for 24 h. As shown (Fig. 8), the burst duration and average number of spikes per burst are both abnormally elevated in *p53^f/+-Emx-Cre+^* cultures as compared with what we observed in Figures 1, 6 where burst activity shows no difference in WT or *p53^f/+-Emx-Cre−^*cultures following the treatments of DHPG. Importantly, this abnormally elevated burst activity can again be corrected by treatments of MK-2206 (1 μM) during the last 1 h of DHPG treatments. Treatments of MK-2206 did not produce significant effects in *p53^f/+-Emx-Cre−^* cultures (Extended Data Fig. 7-2*B*). These results suggest that a potential desensitization or homeostatic reduction of burst activity following chronic activation of Gp1 mGluRs, as we observed in WT cultures (Fig. 2), requires p53-mediated inactivation of Akt.

## Discussion

**Figure 9.**
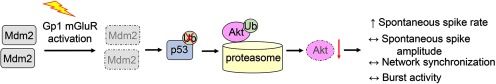
A working model for Mdm2, p53, and Akt in chronic activation of Gp1 mGluRs-induced refinement of neural network activity. Chronic activation of Gp1 mGluR leads to reduction of Mdm2, elevation of p53 and proteasomal degradation of Akt. Subsequently, neural network activity is adjusted, including elevation of spontaneous spike frequency and maintenance of spontaneous spike amplitude, network synchronization, and burst activity at the baseline.

Our study revealed that chronic activation of Gp1 mGluRs in cortical neuron cultures triggers Mdm2-p53 signaling-dependent downregulation of Akt, and subsequently refines the neural network activity (Fig. 9). Specifically, on chronic activation of Gp1 mGluRs, the spontaneous spike frequency is able to be elevated while the spontaneous spike amplitude, neural network synchrony and burst firing activity are able to be maintained at the baseline. Because a previous study has shown that burst firing can be elevated on acute activation of Gp1 mGluRs (Liu et al., 2017), our current study suggests a desensitization or homeostatic reduction of neural network burst activity is likely occurring after chronic activation of Gp1 mGluRs, and p53-dependent inactivation of Akt is required for this process. However, in regards to spontaneous spike frequency and neural network synchrony, p53-dependent inactivation of Akt instead functions to prevent a reduction of those network properties following chronic activation of Gp1 mGluRs. Because our data indicated that DHPG is no longer active after being administered for 24 h, the effects on spontaneous spike frequency and neural network synchrony are likely a result of a plasticity event. This is similar to the findings from a previous study showing that DHPG triggers a long-term increase in neuronal excitability that persists hours after DHPG washout (Bianchi et al., 2009). Although all these results point to a critical role for inactivation of Akt in maintaining neural network activity or excitability, the molecular mechanisms underlying the changes in different network properties, such as spontaneous spike frequency and burst activity, are likely different. Because all these network properties contribute one way or the other to the circuit excitability, evaluating the effects of chronic activation of Gp1 mGluRs at the single cell level would further illustrate the ionic mechanisms underlying our observations at the network level, and can be a future direction. Furthermore, because the network properties are potentially different in cultured versus in intact brains, future work would be needed to validate our observations in living animals *in vivo*.

Our results suggest that downregulation of Mdm2 is the key to elevate p53 and to achieve the changes of neural network properties following chronic activation of Gp1 mGluRs. Two molecules known to be activated by Gp1 mGluRs potentially contribute to the downregulation of Mdm2: Anaphase-promoting complex/cyclosome (APC), which directly ubiquitinates Mdm2 (He et al., 2014; Huang et al., 2015); and casein kinase I (CK1), which phosphorylates Mdm2 to facilitate ubiquitination and proteasomal deposition of Mdm2 (Sakaguchi et al., 2000; Liu et al., 2001; Huart et al., 2009). Because both CK1 and APC have been linked to hyperexcitability and epilepsy (Howlett et al., 2013; Kowalski et al., 2014; Rodriguez et al., 2019), based on our study, Mdm2-p53 signaling perhaps functions downstream of CK1 and/or APC to mediate excitability. With the existing tools and deep knowledge of all these proteins in the field of cancer biology, we expect a future work on this topic to quickly illustrate a novel signaling network by which Gp1 mGluRs modulate neural network activity and neuronal excitability.

Evidence suggests that many of Gp1 mGluR-mediated neuronal plasticity mechanisms require *de novo* protein translation (Niere et al., 2012; Jakkamsetti et al., 2013; Di Prisco et al., 2014). This translation often occurs rapidly in dendrites or near synapses for those proteins necessary for local plasticity (Waung et al., 2008; Waung and Huber, 2009). It remains unclear whether the effects from chronic activation of Gp1 mGluRs that we observed on neural network activity also require protein translation. In order to answer this question, we would need to determine at what time point the acute DHPG effect is transitioned into the chronic DHPG effect so a translational inhibitor can be employed to test the necessity of protein translation during chronic DHPG administration without interfering with the protein translation during the acute DHPG administration. A detailed time course experiment would be necessary to address this issue. A recent study showed that p53 is involved in endoplasmic reticulum (ER) stress-dependent translational control (Liu et al., 2019), indicating a possibility that chronic activation of Gp1 mGluRs may modulate translation through p53. Because both elevation of p53 and inactivation of Akt can be predicted to repress translation based on several previous studies (Hou and Klann, 2004; Liu et al., 2017), chronic activation of Gp1 mGluRs is likely to trigger translation repression. Since protein translation is often correlated with neural network activity and neuronal excitability (Zhang et al., 2012; Higashimori et al., 2013), we would predict that the changes of neural network properties following chronic activation of Gp1 mGluRs may be contributed by reduced protein translation. We plan to validate this hypothesis in the future.

Many neurologic and psychiatric disorders are associated with aberrant activity and/or expression levels of Gp1 mGluRs, such as FXS. FXS patients and the disease model of FXS, the *Fmr1* KO mouse, exhibit multiple symptoms associated with neuronal and circuit hyperexcitability, such as sensory hypersensitivity, social anxiety and seizures (Brennan et al., 2006). It is commonly accepted that exaggerated downstream signaling of Gp1 mGluRs, particularly mGluR5, contributes to the hyperexcitability in FXS (Guo et al., 2015, 2016). Based on our current study, it is possible that exaggerated mGluR signaling is promoting p53 expression in FXS. This prediction is supported by other previous studies showing impaired p53 ubiquitination in *Fmr1* KO mice (Jewett et al., 2018; Lee et al., 2018). Because many drugs related to p53 signaling are currently available, with many approved for clinical trials, our current work may suggest the inhibition of p53 as a way to alleviate hyperexcitability in FXS. Future work will be crucially needed to explore this area.

## Synthesis

Reviewing Editor: Quentin Pittman, University of Calgary Decisions are customarily a result of the Reviewing Editor and the peer reviewers coming together and discussing their recommendations until a consensus is reached. When revisions are invited, a fact-based synthesis statement explaining their decision and outlining what is needed to prepare a revision will be listed below. The following reviewer(s) agreed to reveal their identity: Christina Gross.

This manuscript presents novel and original data suggesting that alterations in neuronal network activity of primary cultured neurons after prolonged exposure to the mGlu1/5 antagonist DHPG are mediated by p53-dependent degradation of Akt. This manuscript uses multi-electrode arrays, western blotting and qRT-PCR to support their conclusions. These results are interesting because pathologic activation of mGlu1/5 has been shown in several neurological disorders and identifying the molecular mechanisms involved is important. The study is limited by the fact that network activity is only assessed in dissociated primary neurons without confirmation in an in vivo system, and by the fact that “chronic” mGlu1/5 activation is only achieved by treatment with the drug DHPG, and it is unclear how stable the drug is over time.

Major comments:

1) A major point of this manuscript is the chronic activation of mGluR1/5 but the authors do not provide a thorough analysis that the receptors are indeed chronically activated in their system. How long is DHPG stable at 37C, in other words, how long are mGluRs active? The authors are urged to perform a time course after DHPG or why did they choose to test 24 hours after addition of DHPG? Justification through literature, if available, or by adding a time course including time points before and after 24 hours would be helpful to understand the rationale and verify their model. In Fig. 3, the authors show that Akt or ERK signaling in their cultures are unchanged. Do they think that DHPG is still active? The authors argue that downregulation of Akt is one of the mechanisms in response to chronic DHPG exposure, but this should be explained better.

Along a similar line, previous work by Wong and colleagues has demonstrated that 1 hour application of DHPG to hippocampal CA3 can evoke a long-term increase in excitability, what they have termed an epilepsy, that lasts after the DHPG is washed out. It may the likely the authors are studying a similar phenomenon. The authors should reference the Wong work. Thus, it would be good to know if these increases in excitability are a form of plasticity or if this is due to lingering DHPG in the culture. Recording after DHPG washout would address this question and put into context of previous work.

2. The authors results indicate a complex mechanism for down regulation of Akt which involves p53 and there is still much that is not known. The authors show that MG132 blocks the decrease in Akt, but this result indicates that something must be degraded by the proteasome for Akt decreases, which could be Mdm2. To better support their conclusion that Akt is degraded by the proteasome, they need additional evidence, such as demonstrating ubiquitination of Akt in response to DHPG. Moreover, important controls validating their findings with the Akt inhibitor are missing. To support the specificity of proteasomal degradation towards Akt, it would be important to show the effect of MG132 on ERK (Fig. 3C).

3) A better way to analyze the effects of DHPG on emx-p53 mouse cultures and their littermates would be to analyze them on one gel using ANOVA testing to correct for multiple testing (Fig. 5B). The authors also should include a control showing how chronic DHPG affects p53 levels in these mice.

4) The authors’ conclusion that Akt is overactive in p53 knockdown neurons would be further validated if they would include data showing that Akt downstream signaling is indeed increased compared to the control neurons.

5) The authors should move extended data Fig.6-1 to the main manuscript and should show the effect of MK-226 on the control mice (spike frequency, synchrony and bursts, Fig. 6 and 7). With the currently available data it is unclear if the Akt inhibitor has a specific or general effect.

6) The authors find that in the p53 het cultures, DHPG results in decreased excitability. Because of the role of p53 in cell viability, it would be important to show that the decreases in spiking frequency are not due a decrease in cell viability or number in these cultures.

7) To better link the biochemical mechanism with the increase in excitability, the authors should test MG132 on the increases in excitability with chronic DHPG.

8) Previous work, referenced by the authors, demonstrate that 30min of DHPG downregulates Mdm2 and increases excitability through a protein synthesis-dependent mechanism using the same culture system (Liu et al., 2017). Is this is the same mechanism here? Does cycloheximide block? This experiments is not absolutely necessary, but demonstrating that a proteostasis is required (degradation and synthesis) would elevate the impact of the work.

Other comments:

1) The authors should show dot plots instead of just bar diagrams to enable a better assessment of their data.

2) The manuscript relies on solely on cultured neuronal cultures that have very different network properties than in vivo brain circuits. The significance of this study would be greatly enhanced if similar mechanisms would be shown in vivo in an intact brain. This should be mentioned as a limitation.

3) The visual abstract could be a bit more detailed, including ubiquitination and degradation by proteosome, where known.

4) Introduction, line 46: The authors may wish to also cite Ronesi, Huber et al., 2012.

5) The authors should limit their statements about novelty of the results.

6) The authors state that the threshold to count an event as a spike was +/- 6 standard deviations. It would be helpful to justify why this is an appropriate threshold. Did the authors take shape of the spikes into account?

7) In the beginning of the results, we suggest including a statement that DHPG was left in the culture and not washed out.

8) Line 197: this should be “widely” not “wildly”.

9) There are a few grammatical errors and typos that should be corrected.

10) In figure 8 and related discussion, the term “refinement of neural network activity” should be explained.

11) The authors do not indicate the source of the Akt inhibitor or how it is prepared.
