# Supplementary figures and images for: Chronic Activation of Gp1 mGluRs Leads to Distinct Refinement of Neural Network Activity through Non-Canonical p53 and Akt Signaling
Source: eNeuro. 2020 Mar 27;7(2):ENEURO.0438-19.2020. doi: 10.1523/ENEURO.0438-19.2020 (PMC7218008; doi:10.1523/ENEURO.0438-19.2020)

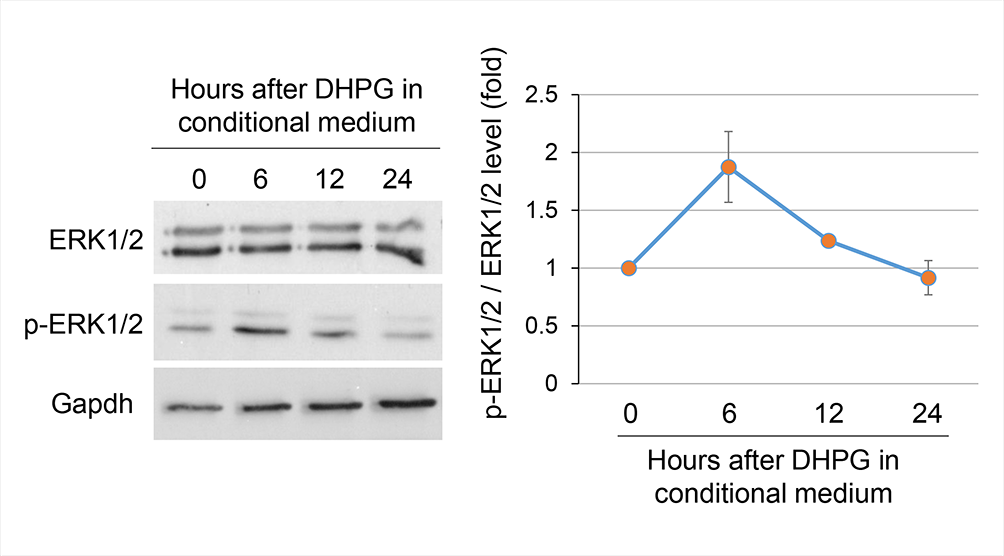

Supplement: Extended Data Figure 1-1 — Time course of DHPG activity in cultures. Representative Western blottings of ERK, p-ERK, and Gapdh, and their quantification from WT cortical neuron cultures treated with conditional medium collected from cultures that were administered with DHPG for 0, 6, 12, or 24 h (n = 4). Download Figure 1-1, TIF file [file enu-eN-NWR-0438-19-s01.tif]

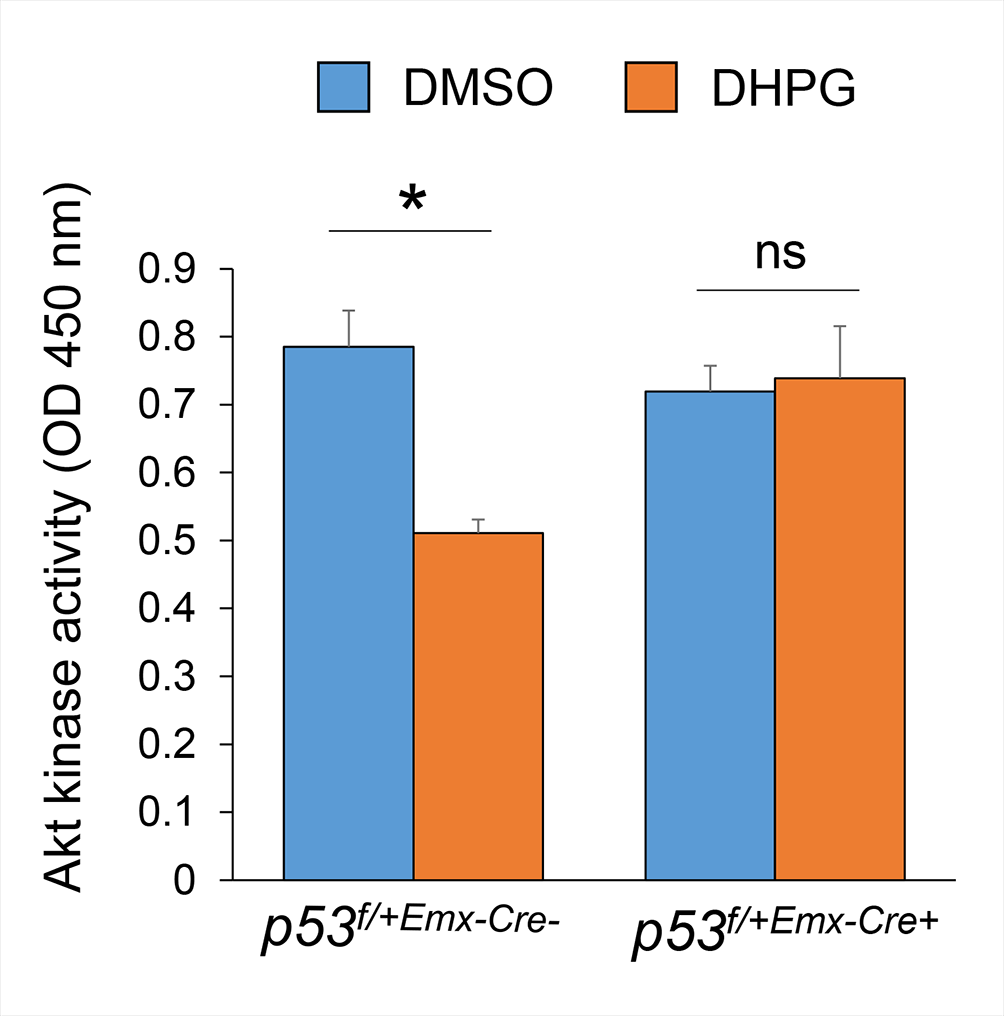

Supplement: Extended Data Figure 3-1 — DHPG treatment for 24 h triggers p53-dependent reduction of Akt kinase activity. Quantification of Akt kinase activity from DMSO- or DHPG-treated p53f/+−Emx1-Cre− or p53f/+−Emx1-Cre+ cortical neuron cultures (n = 6). A two-way ANOVA with Tukey’s test was used. Data are represented as mean ± SEM with *p < 0.05, ns: non-significant. Download Figure 3-1, TIF file. [file enu-eN-NWR-0438-19-s02.tif]

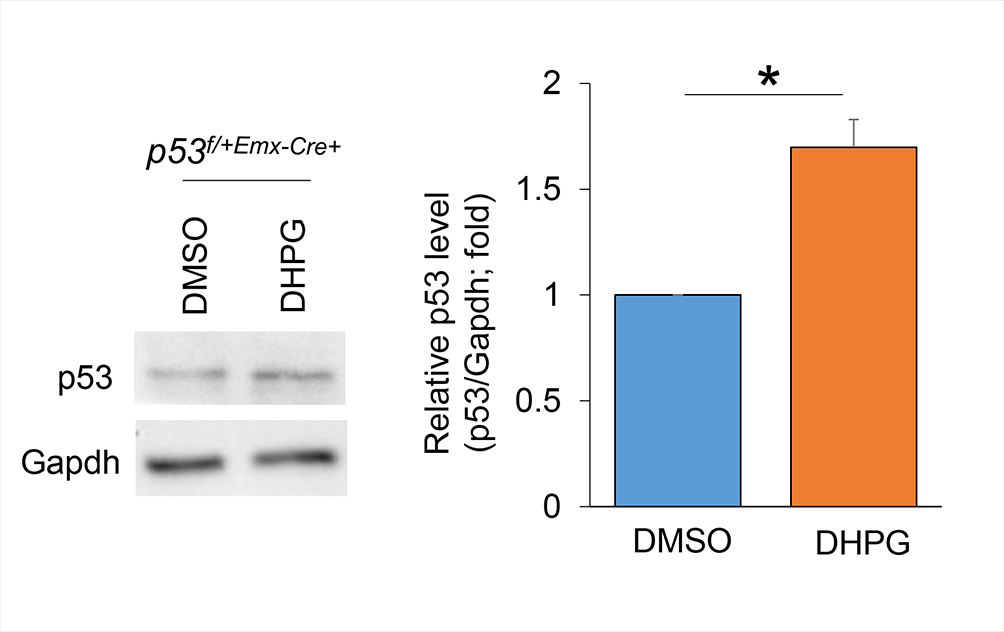

Supplement: Extended Data Figure 5-1 — DHPG treatment for 24 h elevates p53 in p53f/+−Emx1-Cre+ cultures. Representative Western blottings of p53 and Gapdh from p53f/+−Emx1-Cre+ cortical neuron cultures treated with DMSO or DHPG (100 μM) for 24 h at DIV14 (n = 4). Student’s t test was used. Data are represented as mean ± SEM with *p < 0.05. Download Figure 5-1, TIF file. [file enu-eN-NWR-0438-19-s03.tif]

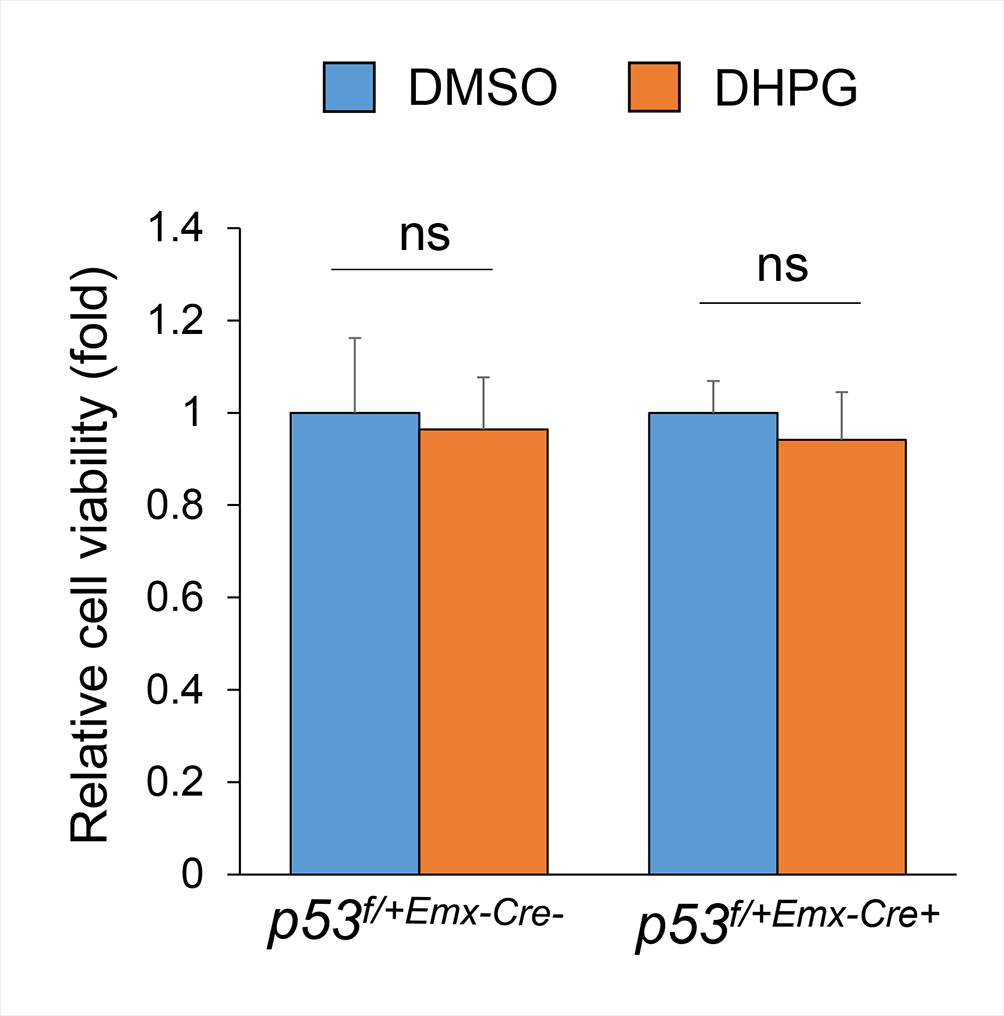

Supplement: Extended Data Figure 7-1 — DHPG treatment for 24 h does not affect cell viability. Quantification of cell viability from DMSO- or DHPG-treated p53f/+−Emx1-Cre− or p53f/+−Emx1-Cre+ cortical neuron cultures (n = 5). A two-way ANOVA with Tukey’s test was used. Data are represented as mean ± SEM with ns: non-significant. Download Figure 7-1, TIF file. [file enu-eN-NWR-0438-19-s04.tif]

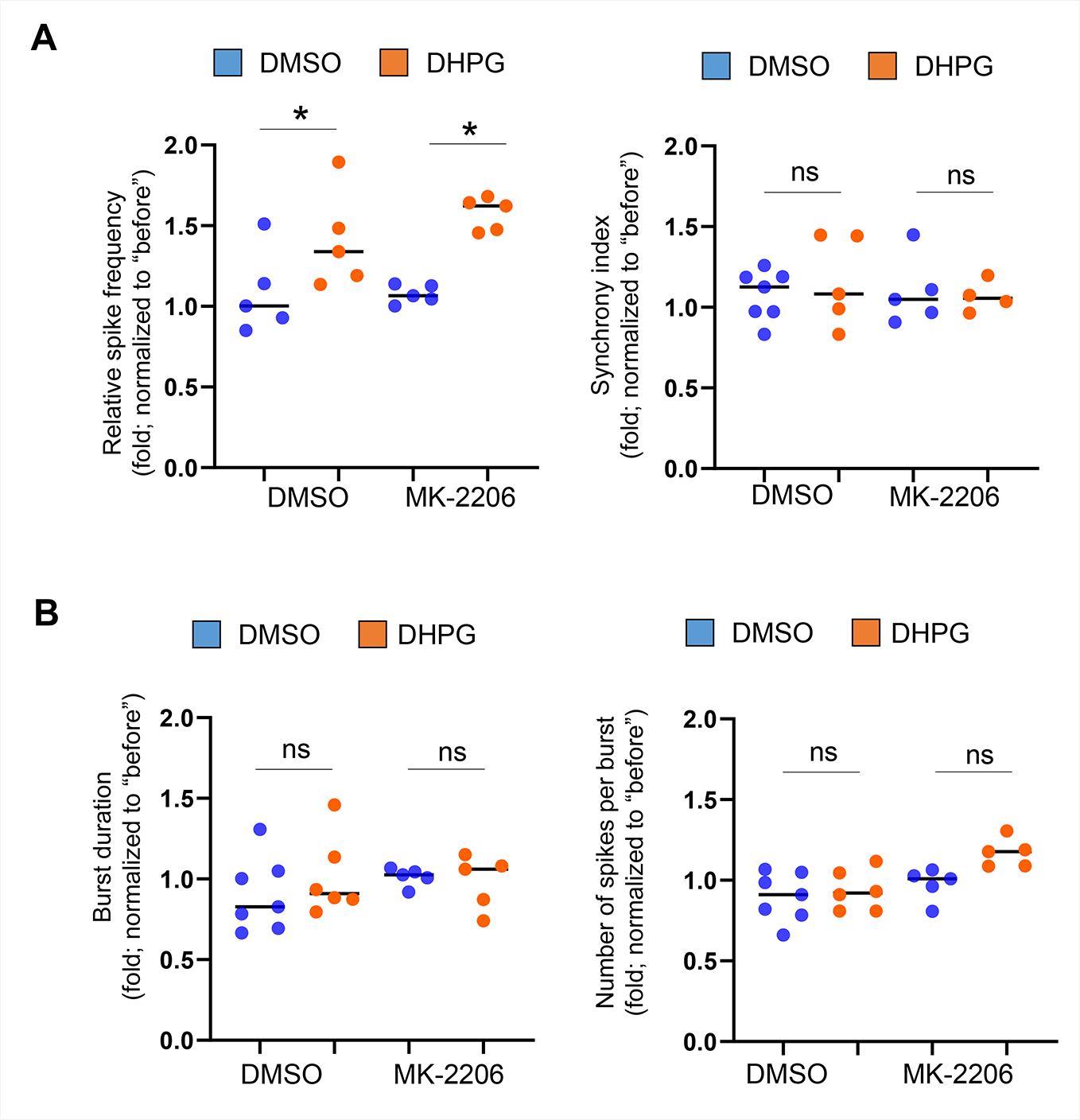

Supplement: Extended Data Figure 7-2 — Inhibition of Akt does not affect basal neural network activity in control, p53f/+−Emx1-Cre−, cultures. A, B, Quantification of relative spontaneous spike frequency (A, left), synchrony (A, right), burst duration (B, left) and relative number of spikes per burst (B, right) from p53f/+−Emx1-Cre− cortical neuron cultures treated with DMSO, DHPG (100 μM), DMSO+MK-2206 (1 μM), or DHPG+MK-2206 at DIV14. The analysis was done by comparing “after treatment” to “before treatment” of the same cultures during the 15-min recordings (n = 5–7 independent cultures). A two-way ANOVA with Tukey’s test was used. Data are represented as mean ± SEM with *p < 0.05 and ns: non-significant. Download Figure 7-2, TIF file. [file enu-eN-NWR-0438-19-s05.tif]
